# Transcriptomes of soybean roots and nodules inoculated with *Sinorhizobium fredii* with NopP and NopI variants

**DOI:** 10.1038/s41597-024-03964-z

**Published:** 2024-10-18

**Authors:** Kejing Fan, Zhixia Xiao, Liping Wang, Wai-Lun Cheung, Fuk-Ling Wong, Feng Zhang, Man-Wah Li, Hon-Ming Lam

**Affiliations:** School of Life Sciences and Centre for Soybean Research of the State Key Laboratory of Agrobiotechnology, The Chinese University of Hong Kong, Shatin, Hong Kong SAR P. R. China

**Keywords:** Agriculture, Rhizobial symbiosis

## Abstract

The major crop, soybean, forms root nodules with symbiotic rhizobia, providing energy and carbon to the bacteria in exchange for bioavailable nitrogen. The relationship is host-specific and highly host-regulated to maximize energy efficiency. Symbiotic nitrogen fixation (SNF) is greener than synthetic fertilizer for replenishing soil fertility, contributing to yield increase. Nodulation Outer Protein P (NopP) and NopI of the type 3 secretion system (T3SS) of the rhizobium determine host specificity. *Sinorhizobium fredii* CCBAU25509 (R2) and CCBAU45436 (R4) have different NopP and NopI variants, affecting their respective symbiotic compatibilities with the cultivated soybean C08 and the wild soybean W05. Swapping the *NopP* variants between R2 and R4 has been shown to switch their compatibility with C08 with the *rj2/Rfg1* genotype. To understand the effects of Nops on host compatibility, analyses on the transcriptomic data of W05 roots and nodules inoculated with *S. fredii* strains containing Nop variants uncovered many differentially expressed genes related to nodulation and nodule functions, providing important information on the effects of Nops on hosts and nodules.

## Background & Summary

Soybean (*Glycine max*) is an important cash crop and leads the oilseed production in the 2022/2023 crop year worldwide. Soybean seeds contain roughly 18% oil and 38% protein^1^, and are commonly used for human food and animal feed. Soybean establishes symbiosis with soil bacteria (rhizobia) to form root nodules where the rhizobium carries out symbiotic nitrogen fixation (SNF). SNF is an essential biological process and the initial stage of the nitrogen cycle, where nitrogen in the atmosphere is assimilated into ammonia at the expense of sugars supplied by the host. SNF could significantly reduce the usage of synthetic nitrogen fertilizer, the production of which is highly energy intensive with high carbon emission. SNF fulfills 40 to 70% of the plant’s total nitrogen demand, depending on the growing conditions and the compatibility between host plants and rhizobia^2,3^. Therefore, SNF is critical for soybean growth and yield.

The symbiosis between legumes and rhizobia depends on molecular signals and determining factors produced by both symbiotic partners. Among these factors, rhizobial Nodulation Outer Proteins (Nops) are critical in determining host specificity. Nops are secreted into the host plant cells through the type III secretion system (T3SS)^4^. T3SS is common to bacterial pathogens of plants that directly delivers effectors into the cytoplasm of host plant cells, thereby helping bacteria evade the host immune response, by altering host signaling pathways and suppressing host defense genes^4,5^. Nops have dual effects on symbiosis, by promoting symbiosis with one legume while impairing the symbiotic process with another^6^. For example, NopP from both *Bradyrhizobium* and *Sinorhizobium* was responsible for the symbiotic incompatibility with soybean carrying different alleles of *Rj2/Rfg1*^4,7^. An incompatible *Rj2/Rfg1*-NopP pair suppressed the formation of the infection thread at two days post-inoculation by activating the host defense gene *PR-2*^4^. Consequently, the soybean host rejected the rhizobial infection, leading to failure in nodule formation.

In our previous work, the soybean cultivar C08 carrying the *rj2/Rfg1* allele was found to be incompatible with *Sinorhizobium fredii* CCBAU25509 (R2) but compatible with CCBAU45436 (R4)^7,8^. The incompatibility was due to the sequence variations in NopP between R2 and R4. NopI, the paralog of NopP, also exhibited sequence variations between R2 and R4, although NopI did not appear to contribute to host specificity^9^.

**Fig. 1:**
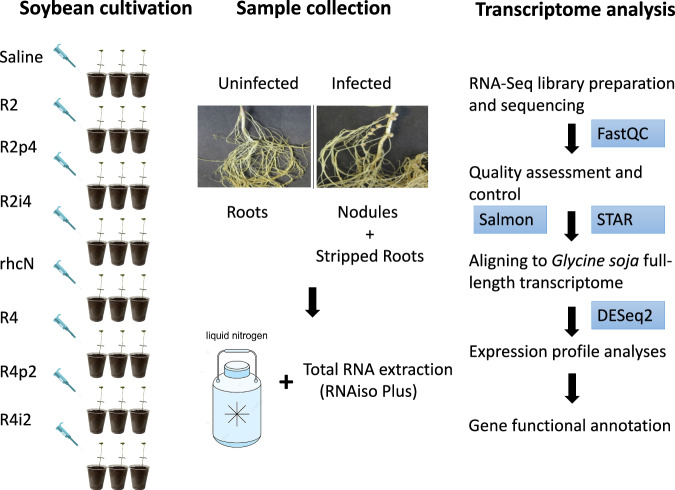
Experimental design of this study. To detect the transcriptome profile changes in wild soybean (W05) roots and nodules inoculated with different strains of *Sinorhizobium fredii* versus uninfected roots, stripped roots and nodules were harvested 28 days after inoculation. For each strain, there were three biological replicates, each consisting of three individual plants harvested together as one sample. Three biological replicates per treatment were used for transcriptome sequencing. Only the clean reads were used for analyses. R2 and R4 are two wildtype strains of *S. fredii* with different host compatibilities. R2p4, R2 with NopP from R4 swapped in; R2i4, R2 with NopI from R4 swapped in; R4p2, R4 with NopP from R2 swapped in; R4i2, R4 with NopI from R2 swapped in; rhcN, R2 T3SS mutant.

In this research, to investigate the functions of NopP and NopI in effecting host compatibility and nodule functions, we generated transcriptomic data on a compatible soybean host inoculated with different NopP and NopI variants. The wild-type R2 and R4 strains of *S. fredii* were used as background and their respective *NopP* and *NopI* genes were swapped between them. To be specific, NopP- and NopI-swapped strains of R2 and R4, along with their corresponding wild-type strains and an R2 T3SS mutant (rhcN), were inoculated into the compatible wild soybean W05 carrying the *rj2/rfg1* allele. Transcriptomic data from W05 uninfected roots, stripped infected roots and nodules were collected and analyzed. A flowchart of the experimental design is presented in Fig. 1. The purpose of the present study was to analyze the transcriptional changes in soybean nodules attributable to the NopP and NopI variants, to further the investigation into the roles of Nops in mature soybean nodules.

## Methods

### *T3SS, NopP-* and *NopI*-swapped mutants of *Sinorhizobium fredii* CCBAU25509 (R2) and CCBAU45436 (R4)

The R2 T3SS mutant (*rhcN::Tn5*, rhcN thereafter) and *NopP*-swapped mutant of R2 (R2p4) and R4 (R4p2) were constructed in our previous work^7^. For the NopI mutants, the *NopI* coding sequences with ~1 kb upstream/downstream were amplified from the genomic DNA of R2 and R4, respectively, and inserted into pK18mobsacB (ATCC [American Type Culture Collection], Cat.# 87097). The protocol for swapping of *NopI* genes was described in our previous study^7^. The primers for generating and screening the mutants can be found in Supplementary Table 1.

### Cultivation of bacteria and plants

Seeds of the wild soybean (*Glycine soja*) accession W05^10^ were surface-sterilized with chlorine gas for 16 h and germinated in sterilized vermiculite in the greenhouse in total darkness^11^. Four days after sowing, seedlings were transferred into individual pots supplemented with 1X B & D solution according to a previous report^12^.

The *Sinorhizobium fredii* strains (R2, R2p4 [R2 with NopP from R4], R2i4 [R2 with NopI from R4], rhcN [an R2 T3SS mutant], R4, R4p2 [R4 with NopP from R2] and R4i2 [R4 with NopI from R2]) were cultured in TY medium containing 5 g L^−1^ tryptone, 3 g L^−1^ yeast extract and 0.88 g L^−1^ CaCl_2_ at 28 °C with shaking at 180 rpm for 40 h^13^. Before inoculation, the rhizobium cultures were harvested by centrifugation at 1,500 rpm and resuspended in sterile saline solution (0.9% NaCl w/v) to a final OD_600_ = 0.2. One milliliter of the bacterial suspension was inoculated onto the root of each seedling in the vermiculite. Control plants were prepared following the same procedure but inoculated with sterile saline only. At 28 days after inoculation, nodules and roots were harvested separately and frozen immediately in liquid nitrogen. The samples were then stored at −80 °C for RNA extraction later.

### RNA extraction and RNA-Seq

Twenty-four root samples (three uninfected roots and 21 stripped roots) and 21 mature nodule samples, with three biological replicates per treatment/strain-Nop combination per tissue, were used for total RNA extraction using RNAiso Plus (Takara, Cat.# 9108/9109). Total RNAs were sent to Novogene Co, Ltd. for RNA sequencing. Strand-specific polyA-enriched libraries were sequenced on an Illumina NovaSeq6000 platform to generate at least 50 million PE150 reads for each library.

### Analysis of differential gene expression and quantification of gene expressions

The nf-core RNAseq version 3.3 pipeline (https://github.com/nf-core/rnaseq) was employed for the processing and analysis of raw RNA sequencing data. This pipeline utilizes STAR, DESeq2, and Salmon to generate gene counts and conducts comprehensive quality control assessments. Raw RNA sequencing data, including 45 sequencing data files, and the wild soybean W05 reference genome^10^, were utilized. Within the nf-core/rnaseq pipeline, quality control procedures involved the utilization of FastQC version 0.11.9 (https://www.bioinformatics.babraham.ac.uk/projects/fastqc/) for adapter trimming, filtering of low-quality sequences, and the removal of bases with a Phred quality score below 20. Subsequently, alignment was performed using STAR version 2.7.6a^14^ to map the sequencing reads to the reference genome. The expression levels were estimated using Salmon version 1.4.0^15^, and the gene expression matrix was generated. To evaluate sample similarities based on gene expression profiles, a sample similarity clustering heatmap was generated using the expression data processed through the nf-core/rnaseq pipelineIn R language. Euclidean distance was used to calculate the similarity between samples, and R package pheatmap was used to visualize the clustering heatmap.

DESeq2 version 1.28.0^16^ was employed to detect differential gene expression. The negative binomial distribution model was utilized to characterize the distribution properties of RNA-seq data and assess gene expression changes under varying conditions. Significantly altered gene expression levels were identified using the criteria of log_2_(fold change) ≥ 1 and FDR (False Discovery Rate) ≤ 0.05.

## Data Records

The raw data used in this work was submitted to the Sequence Read Archive (SRA) at the National Center for Biotechnology Information (NCBI) database with the accession number PRJNA1112908.

This data set consists of 45 sample files, which are classified into the following three main categories according to the *Sinorhizobium fredii* strains used (R2, R4 and uninfected control):

1. Soybean W05 infected with R2, R2p4, R2i4 and rhcN, including stripped root and nodule tissues, each with three biological replicates, named as R2/R2p4/R2i4/rhcN_root/Nod _replicate_number_r1/r2/r3^17^.
2. Soybean W05 infected with R4, R4p2 and R4i2, including stripped root and nodule tissues, each with three biological replicates, named as R4/R4p2/R4i2_root/Nod_r1/2/3^17^.
3. Soybean W05 mock-infected with saline, including uninfected root tissues only, with three biological replicates, named as UNino_root_r1/2/3^17^.

The processed data files, gene_counts.tsv and gene_tpm.tsv, were deposited on Gene Expression Omnibus (GEO) at the NCBI database with the accession number GSE274768^18^.

## Technical Validation

### Data quality assessment

The symbiosis between soybean and rhizobium can significantly enhance soybean growth under low nitrogen conditions and improve the plant nitrogen content, making leaves greener. To investigate the effect of swapping NopP and NopI between R2 and R4, we inoculated wild soybean W05 with saline (uninoculated), R2, R2p4, R2i4, rhcN, R4, R4p2 and R4i2. The root and nodule samples were harvested to do transcriptome sequencing.

**Fig. 2:**
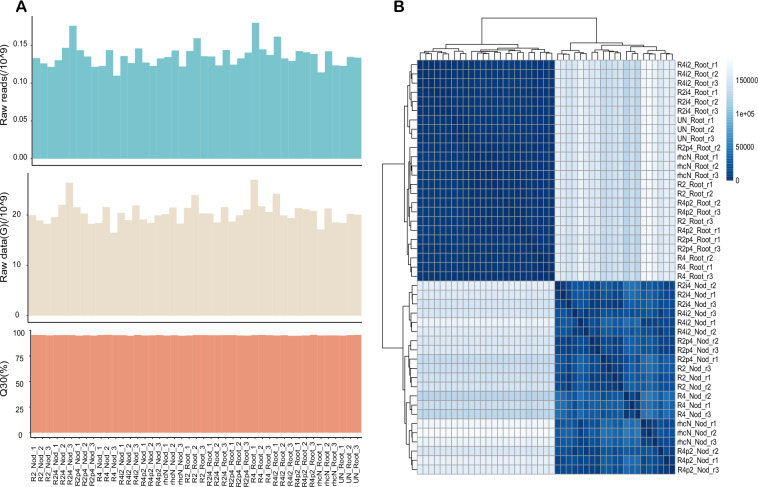
Technical validation of the transcriptomic data. (**A**) Overview of the RNA-seq data. Raw reads (top panel), raw data information (second panel), Q30 (%) (third panel) and GC (%) (bottom panel) for each RNA-seq library were plotted. (**B**) A heatmap showing the correlation coefficients of the scaled expression levels among all sample pairs. R2 and R4 are two wildtype strains of *S. fredii* with different host compatibilities. R2p4, R2 with NopP from R4 swapped in; R2i4, R2 with NopI from R4 swapped in; R4p2, R4 with NopP from R2 swapped in; R4i2, R4 with NopI from R2 swapped in; rhcN, R2 T3SS mutant; Unino, uninoculated; Nod, nodule; r1, r2 and r3, replicates 1, 2 and 3.

The quality of the RNA-seq data for each sample is shown in Fig. 2. Briefly, 9.93 Gb of data on average were obtained for each sample (Supplementary Table 2). FastQC was used to confirm the base quality of the sequencing reads. The average high-quality score, Q30 (base error < 0.1%), was 95.27% (Fig. 2A).

To assess the reproducibility of the biological replicates, we used DEseq2 to calculate the correlation of the expression profile from each sample. In general, gene expressions were highly correlated among biological replicates of the same kind of tissue samples (i.e., roots or nodules) (Fig. 2B).

### Differentially expressed gene (DEG) analysis

**Fig. 3:**
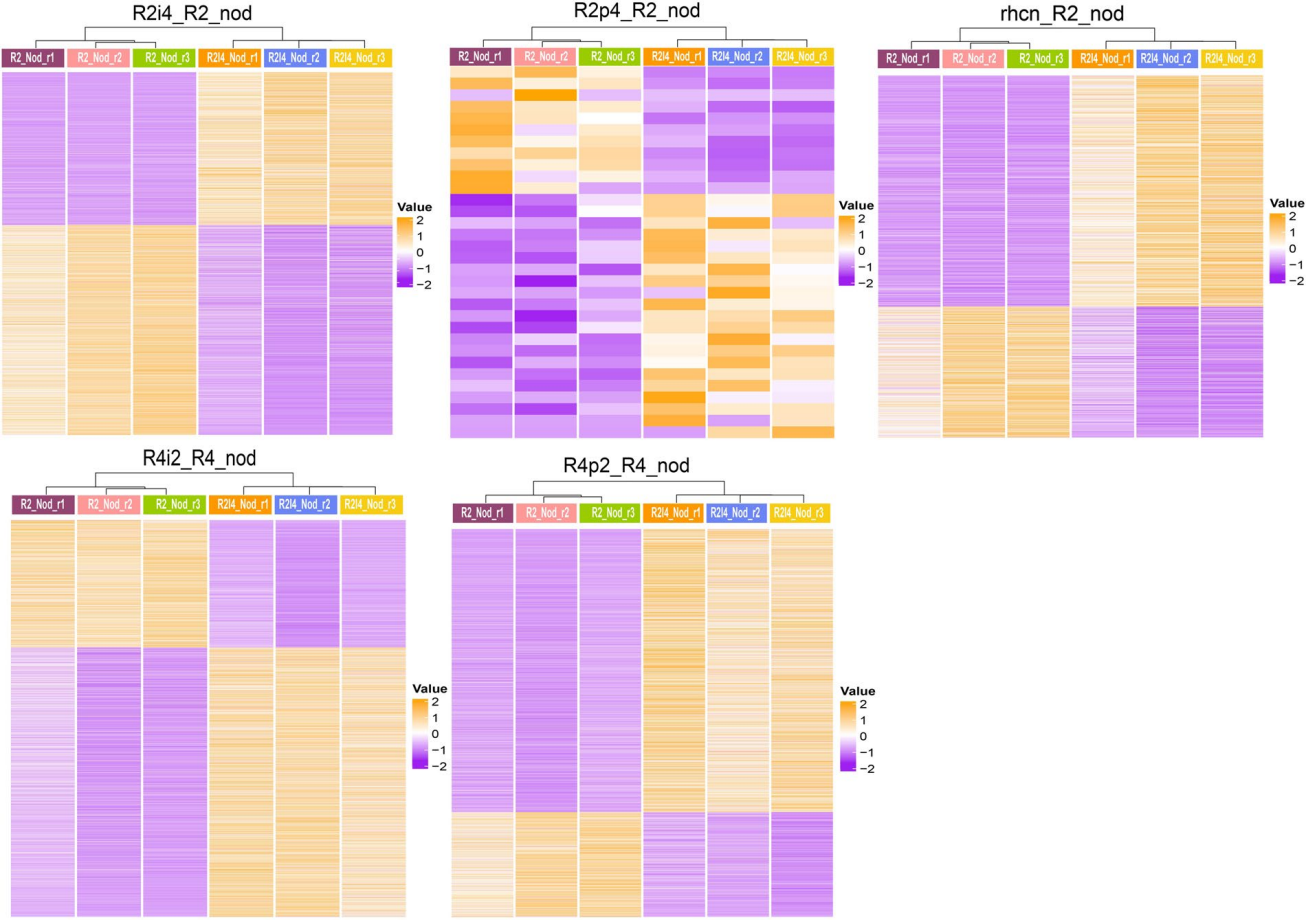
The expression profiles of differentially expressed genes in nodules inoculated with different strains of *Sinorhizobium fredii* carrying variants of NopI and NopP. R2 and R4 are two wildtype strains of *S. fredii* with different host compatibilities. R2p4, R2 with NopP from R4 swapped in; R2i4, R2 with NopI from R4 swapped in; R4p2, R4 with NopP from R2 swapped in; R4i2, R4 with NopI from R2 swapped in; rhcN, R2 T3SS mutant; nod, nodule; r1, r2, r3, replicates 1, 2 and 3. The expression levels of genes [Log_2_(FPKM + 1)] were presented in different colors according to the color key.

Differential expression analyses between samples/treatments were performed with DEseq2 based on the following criteria: log_2_(Fold Change) ≥1 and false discovery rate (FDR) <0.05. Summaries of the comparisons are presented in Table 1, Fig. 3 and Supplementary Figure 1. The differentially expressed genes (DEGs) in nodules are shown in Fig. 3 and those from other tissues in Supplementary Figure 1. To assess the data variation caused by Nops, we compared the samples infected with R2 mutants to those infected with wild-type R2 and uninfected roots (Table 1). Similar comparisons were also made with respect to wild-type and mutated R4 strains (Table 1). Among all the R2 mutant-related samples, infection with R2p4 resulted in the lowest number of DEGs in both roots and nodules, with 71 DEGs in roots and 32 in nodules. On the other hand, infection with R2i4 was associated with the highest number of DEGs (1,083 in stripped roots and 2,386 in nodules). rhcN infection had 15 DEGs in roots and 1,468 DEGs in nodules. R4p2 infection resulted in 333 DEGs in roots and 2,325 in nodules. R4i2 infection was associated with 1,100 DEGs in roots and 2,861 in nodules.

**Table 1:** Numbers of differentially expressed genes identified in each pairwise comparison.

Pairwise comparison	Change in expression	Number of genes
**R2_root_vs_UN_Root**	down	425
**R2_root_vs_UN_Root**	up	652
**R2i4_Nod_vs_R2_Nod**	down	1005
**R2i4_Nod_vs_R2_Nod**	up	1381
**R2i4_root_vs_R2_root**	down	486
**R2i4_root_vs_R2_root**	up	597
**R2i4_root_vs_UN_Root**	down	87
**R2i4_root_vs_UN_Root**	up	179
**R2p4_Nod_vs_R2_Nod**	down	11
**R2p4_Nod_vs_R2_Nod**	up	21
**R2p4_root_vs_R2_root**	down	22
**R2p4_root_vs_R2_root**	up	49
**R2p4_root_vs_UN_Root**	down	157
**R2p4_root_vs_UN_Root**	up	541
**R4_root_vs_UN_Root**	down	282
**R4_root_vs_UN_Root**	up	869
**R4i2_Nod_vs_R4_Nod**	down	919
**R4i2_Nod_vs_R4_Nod**	up	1942
**R4i2_root_vs_R4_root**	down	614
**R4i2_root_vs_R4_root**	up	486
**R4i2_root_vs_UN_Root**	down	96
**R4i2_root_vs_UN_Root**	up	340
**R4p2_Nod_vs_R4_Nod**	down	1697
**R4p2_Nod_vs_R4_Nod**	up	628
**R4p2_root_vs_R4_root**	down	275
**R4p2_root_vs_R4_root**	up	58
**R4p2_root_vs_UN_Root**	down	803
**R4p2_root_vs_UN_Root**	up	766
**rhcN_Nod_vs_R2_Nod**	down	937
**rhcN_Nod_vs_R2_Nod**	up	531
**rhcN_root_vs_R2_root**	down	4
**rhcN_root_vs_R2_root**	up	11
**rhcN_root_vs_UN_Root**	down	211
**rhcN_root_vs_UN_Root**	up	366

Nod, nodule; UN, uninoculated; R2 and R4 are two wildtype strains of *S. fredii* with different host compatibilities. R2p4, R2 with NopP from R4 swapped in; R2i4, R2 with NopI from R4 swapped in; R4p2, R4 with NopP from R2 swapped in; R4i2, R4 with NopI from R2 swapped in; rhcN, R2 T3SS mutant.

## Supplementary information

Supplementary tables

Supplementary Figure 1

## Data Availability

The analyses in this research were performed using a previously reported pipeline^19^. The complete gene expression profiles are listed in Supplementary Sheet 1. The software versions used in the analyses and R scripts for figure preparations are presented in Supplementary File 1.
