# Supplementary material for: Transcriptomes of soybean roots and nodules inoculated with Sinorhizobium fredii with NopP and NopI variants
Source: Sci Data. 2024 Oct 18;11:1146. doi: 10.1038/s41597-024-03964-z (PMC11489703; doi:10.1038/s41597-024-03964-z)
Supplement: Supplementary file 1 — Supplementary tables [file 41597_2024_3964_MOESM1_ESM.docx]

**Supplementary Table 1. Primers used in this research.**

| Primer name | Primer sequence |
| --- | --- |
| NopI exchange fragment (5' homology arm) (with XbaI site) forward | AAATCTAGAGGCACTGTAAAGTTATCAGC |
| NopI exchange fragment (5' homology arm) (with KpnI site) reverse | AAAGGTACCGCGGTTCGCTCCCCAAT |
| NopI exchange fragment (with KpnI site) forward | AAAGGTACCATGTATAGCAGTATCACTGGATCAGC |
| NopI exchange fragment (with EcoRI site) reverse | AAAGAATTCTTAGTCGGCCGGTCCT |
| NopI exchange fragment (3' homology arm) (with EcoRI site) forward | AAAGAATTCGAGGTTCAATAGGGAGCG |
| NopI exchange fragment (3' homology arm) (with PstI site) reverse | AAACTGCAGCTCGCCTTCCCGATGC |
| NopI recombination checking forward | CAATTGGGGAGCGAACCGCGGT |
| NopI recombination checking reverse | GCAATGTGCTTGACATCATCACTAT |

**Supplementary Table 2. Summary of RNA sequencing reads mapping statistics.**

| **Sample**  **(strain_tissue_replicate)** | **clean_reads** | **mapped_reads** | **mapped_percent** |
| --- | --- | --- | --- |
| R2i4_Nod_r1 | 65139836 | 58551827 | 89.89 |
| R2i4_Nod_r2 | 73293842 | 66106641 | 90.19 |
| R2i4_Nod_r3 | 87929776 | 79670292 | 90.61 |
| R2i4_root_r1 | 67563125 | 63462785 | 93.93 |
| R2i4_root_r2 | 61723869 | 57795919 | 93.64 |
| R2i4_root_r3 | 71865342 | 67331807 | 93.69 |
| R2p4_Nod_r1 | 71758042 | 66340918 | 92.45 |
| R2p4_Nod_r2 | 67572854 | 61749785 | 91.38 |
| R2p4_Nod_r3 | 60802563 | 55813062 | 91.79 |
| R2p4_root_r1 | 62233680 | 58121774 | 93.39 |
| R2p4_root_r2 | 66395842 | 63071932 | 94.99 |
| R2p4_root_r3 | 70136686 | 65789493 | 93.8 |
| R2_Nod_r1 | 66517288 | 60668351 | 91.21 |
| R2_Nod_r2 | 63016659 | 57119996 | 90.64 |
| R2_Nod_r3 | 60751637 | 56064793 | 92.29 |
| R2_root_r1 | 71250410 | 66334096 | 93.1 |
| R2_root_r2 | 79680072 | 74482368 | 93.48 |
| R2_root_r3 | 67741847 | 63449824 | 93.66 |
| R4i2_Nod_r1 | 67896872 | 60880614 | 89.67 |
| R4i2_Nod_r2 | 63201932 | 57932714 | 91.66 |
| R4i2_Nod_r3 | 72903077 | 65860830 | 90.34 |
| R4i2_root_r1 | 80725018 | 76936024 | 95.31 |
| R4i2_root_r2 | 66317232 | 62311154 | 93.96 |
| R4i2_root_r3 | 64631265 | 61615114 | 95.33 |
| R4p2_Nod_r1 | 63610623 | 57524929 | 90.43 |
| R4p2_Nod_r2 | 61378711 | 55742431 | 90.82 |
| R4p2_Nod_r3 | 66297938 | 58389120 | 88.07 |
| R4p2_root_r1 | 71180007 | 67721761 | 95.14 |
| R4p2_root_r2 | 70330351 | 67041928 | 95.32 |
| R4p2_root_r3 | 69211215 | 65072087 | 94.02 |
| R4_Nod_r1 | 61314754 | 55483782 | 90.49 |
| R4_Nod_r2 | 71791039 | 65456276 | 91.18 |
| R4_Nod_r3 | 62261957 | 56691231 | 91.05 |
| R4_root_r1 | 89904891 | 84314818 | 93.78 |
| R4_root_r2 | 72349684 | 67817725 | 93.74 |
| R4_root_r3 | 68628087 | 64400782 | 93.84 |
| UN_Root_r1 | 61355643 | 58167681 | 94.8 |
| UN_Root_r2 | 67342348 | 63177310 | 93.82 |
| UN_Root_r3 | 66810468 | 62772655 | 93.96 |
| rhcN_Nod_r1 | 67159971 | 60883179 | 90.65 |
| rhcN_Nod_r2 | 71508640 | 63916557 | 89.38 |
| rhcN_Nod_r3 | 60967665 | 55815848 | 91.55 |
| rhcN_root_r1 | 57076293 | 54147803 | 94.87 |
| rhcN_root_r2 | 70996363 | 66058845 | 93.05 |
| rhcN_root_r3 | 61706506 | 57512017 | 93.2 |

R2 and R4, two wildtype strains of *S. fredii* with different host compatibilities; R2p4, R2 with NopP from R4 swapped in; R2i4, R2 with NopI from R4 swapped in; R4p2, R4 with NopP from R2 swapped in; R4i2, R4 with NopI from R2 swapped in; rhcN, R2 T3SS mutant; Nod, nodule; r1, r2 and r3, biological replicates 1, 2 and 3.
