# Supplementary material for: Transcriptomes of soybean roots and nodules inoculated with Sinorhizobium fredii with NopP and NopI variants
Source: Sci Data. 2024 Oct 18;11:1146. doi: 10.1038/s41597-024-03964-z (PMC11489703; doi:10.1038/s41597-024-03964-z)
Supplement: Supplementary file 2 — Supplementary Figure 1 [file 41597_2024_3964_MOESM2_ESM.docx]

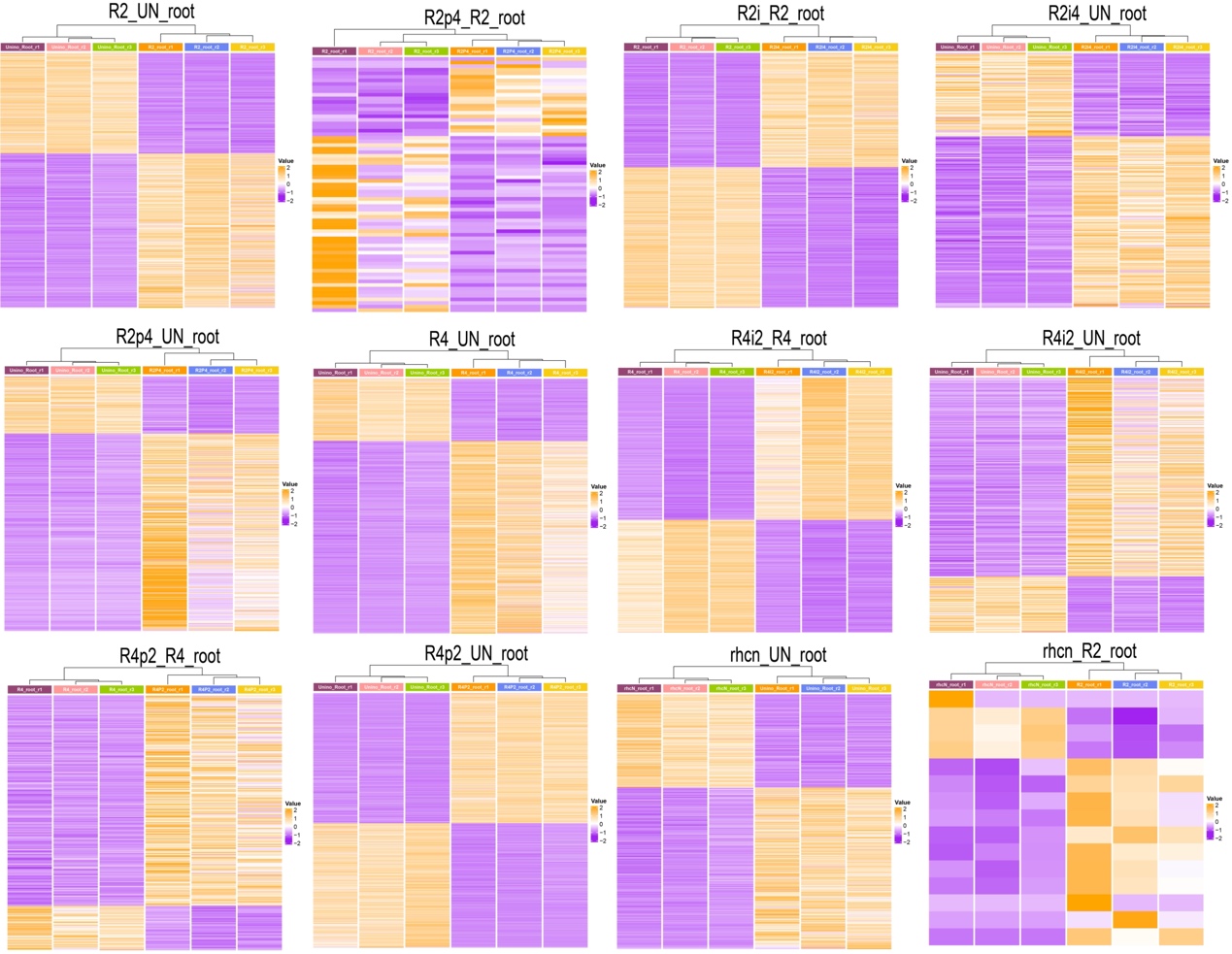


**Supplementary Figure 1. The expression profiles of differentially expressed genes in uninoculated roots and roots inoculated with strains of *Sinorhizobium fredii* carrying different variants of NopI and NopP.** The expression levels of genes [Log_2_(FPKM+1)] were presented in different colors based on the color key. R2 and R4 are two wildtype strains of *S. fredii* with different host compatibilities. R2p4, R2 with NopP from R4 swapped in; R2i4, R2 with NopI from R4 swapped in; R4p2, R4 with NopP from R2 swapped in; R4i2, R4 with NopI from R2 swapped in; rhcN, R2 T3SS mutant; Unino/UN, uninoculated; r1, r2 and r3, biological replicates 1, 2 and 3.
